# Truncation of the TAR DNA-binding protein 43 is not a prerequisite for cytoplasmic relocalization, and is suppressed by caspase inhibition and by introduction of the A90V sequence variant

**DOI:** 10.1371/journal.pone.0177181

**Published:** 2017-05-16

**Authors:** Heike J. Wobst, Louise Delsing, Nicholas J. Brandon, Stephen J. Moss

**Affiliations:** 1 AstraZeneca-Tufts Laboratory for Basic and Translational Neuroscience, Tufts University, Boston, MA, United States of America; 2 AstraZeneca, Discovery Science, Innovative Medicines and Early Development Biotech Unit, Mölndal, Sweden; 3 AstraZeneca, Neuroscience, Innovative Medicines and Early Development, Waltham, MA, United States of America; 4 Department of Neuroscience, Tufts University School of Medicine, Boston, MA, United States of America; International Centre for Genetic Engineering and Biotechnology, ITALY

## Abstract

The RNA-binding and -processing protein TAR DNA-binding protein 43 (TDP-43) is heavily linked to the underlying causes and pathology of neurodegenerative diseases such as amyotrophic lateral sclerosis and frontotemporal lobar degeneration. In these diseases, TDP-43 is mislocalized, hyperphosphorylated, ubiquitinated, aggregated and cleaved. The importance of TDP-43 cleavage in the disease pathogenesis is still poorly understood. Here we detail the use of D-sorbitol as an exogenous stressor that causes TDP-43 cleavage in HeLa cells, resulting in a 35 kDa truncated product that accumulates in the cytoplasm within one hour of treatment. We confirm that the formation of this 35 kDa cleavage product is mediated by the activation of caspases. Inhibition of caspases blocks the cleavage of TDP-43, but does not prevent the accumulation of full-length protein in the cytoplasm. Using D-sorbitol as a stressor and caspase activator, we also demonstrate that the A90V variant of TDP-43, which lies adjacent to the caspase cleavage site within the nuclear localization sequence of TDP-43, confers partial resistance against caspase-mediated generation of the 35 kDa cleavage product.

## Introduction

**Fig 1:**
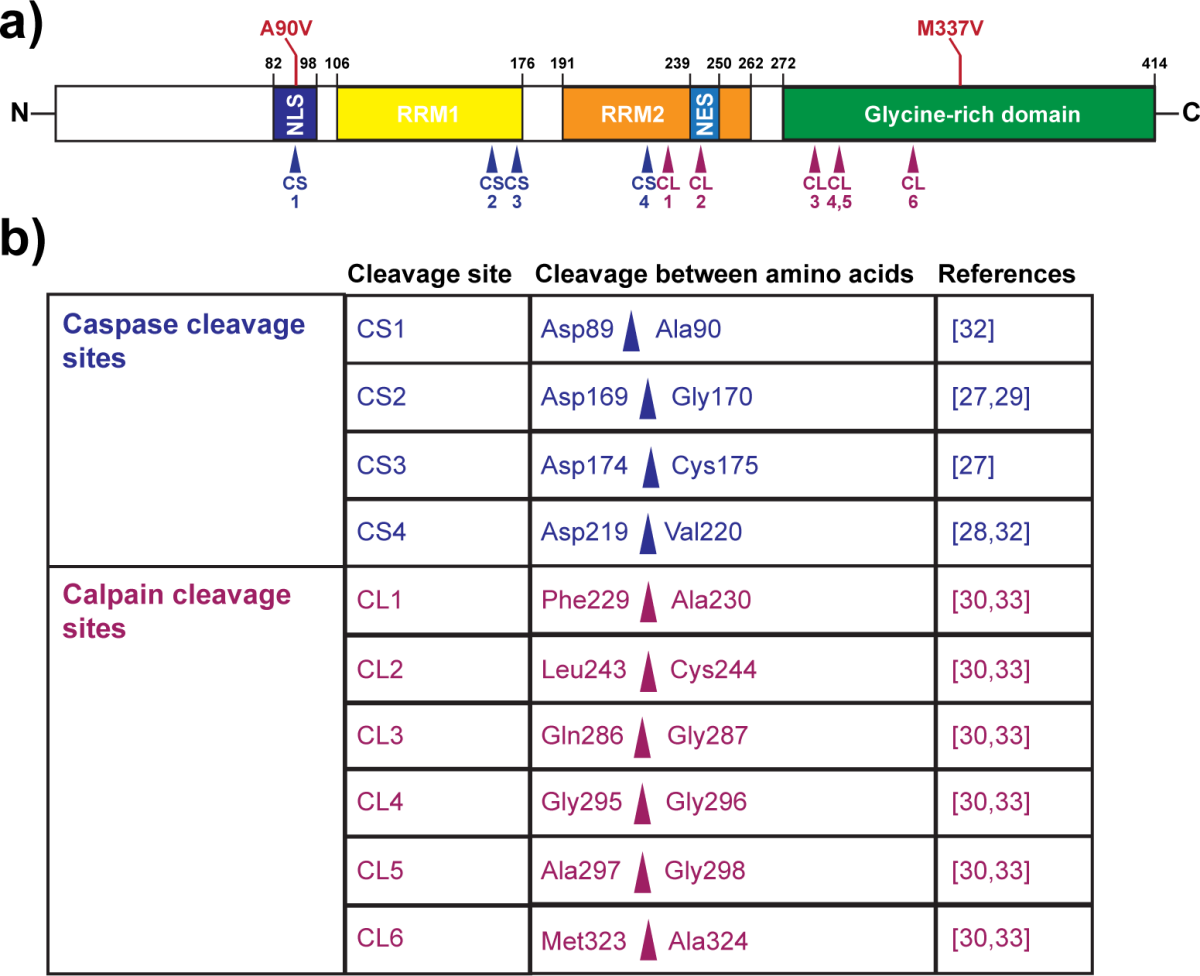
TDP-43 domain structure with caspase and calpain cleavage sites. (A) Schematic of the full-length, 414 amino acid long TDP-43 protein. NLS = nuclear localization signal, RRM1/RRM2 = RNA recognition motifs, NES = nuclear export signal. The A90V variant and the M337V mutation are indicated in red. CS = (putative) caspase cleavage site, CL = (putative) calpain cleavage site. (B) Table of TDP-43 caspase and calpain cleavage sites described in the literature.

TAR DNA-binding protein 43 (TDP-43) is a ubiquitously expressed nuclear RNA- and DNA-binding protein [1, 2]. It is involved in a variety of cellular processes, most of which concern the regulation of RNA, with over 6000 RNA species identified as targets [3]. These processes include splicing of pre-mRNAs, regulation of mRNA stability and turnover as well as mRNA trafficking and sequestration of mRNA into transient structures termed stress granules [3–15]. Furthermore, TDP-43 is a factor in the biogenesis of microRNAs as a component of Drosha and Dicer complexes, and binds to single-stranded DNA as a transcriptional regulator [1, 16–20]. In its carboxy-terminus, TDP-43 contains a glycine-rich, prion-like domain that renders the protein intrinsically prone to aggregation (Fig 1) [21, 22]. Cytoplasmic inclusions of aggregated, ubiquitinated TDP-43 are found in almost all cases of amyotrophic lateral sclerosis (ALS), the most common form of motor neuron disease, as well as in a subtype of frontotemporal lobar degeneration (FTLD) characterized by ubiquitin- and TDP-43-positive inclusions (FTLD-U) [23, 24]. In addition to nuclear-to-cytoplasmic subcellular relocalization, ubiquitination and aggregation of TDP-43 in these diseases, other pathological hallmarks include phosphorylation of TDP-43 at the serine 409/serine 410 site as well as protein truncation, leading to the formation of C-terminal TDP-43 fragments (CTFs) [25, 26]. These C-terminal fragments are generated through the activation of either caspases or calpains. Thus far, a variety of potential caspase and calpain cleavage sites have been described (Fig 1) [27–32].

In brain tissues of FTLD-U patients, CTFs of 20–25 kDa are found in insoluble protein fractions [26], and 35 kDa fragments have been described occasionally in cases of ALS and FTLD-U [32, 33]. Expression of wild-type or mutant TDP-43 in cell and animal models likewise causes the formation of 25 and 35 kDa cleavage products [27, 29, 32, 34–36]. The 35 kDa fragment is generated by cleavage after Asp89, which lies within the bipartite nuclear localization sequence (NLS) of TDP-43, and is specifically generated through activation of the executioner caspases 3 and 7 [32]. Activated caspase-3 has been described in tissues from ALS and FTLD patients [37, 38], suggesting its role in the generation of CTFs in human patients. The clinical relevance of these fragments, however, remains elusive.

Here, we use the sugar alcohol D-sorbitol as a stressor to induce the formation and cytoplasmic accumulation of the 35 kDa fragment of TDP-43 in a cell culture system. We show that the formation of this cleavage product, but not the mislocalization of uncleaved TDP-43 to the cytoplasm, is abrogated by caspase inhibition. We also show that an alanine to valine amino acid substitution at position 90 (A90V) confers partial resistance to caspase cleavage at the Asp89 site.

## Materials and methods

### Cell culture, stress treatments and transfections

HeLa cells (CCL-2, American Type Culture Collection [ATCC]) were cultured in Dulbecco’s modified Eagle medium (DMEM, Thermo Fisher Scientific) supplemented with 10% fetal bovine serum (FBS), 100 u/ml penicillin and 100 μg/ml streptomycin. For immunofluorescence analysis, cells were grown on coverslips in 24-well plates (Cellstar). For stress experiments, cells were treated with either 10 μM N-benzyloxycarbonyl-L-leucyl-L-leucyl-L-leucinal (MG132, Tocris) for 4 h, 1 μM thapsigargin (Sigma) for 60 min, 0.5 mM sodium arsenite (Sigma) for 30 min, 1 mM hydrogen peroxide for 60 min or 0.4 M D-sorbitol (Sigma) for 60 min. Cells were transiently transfected using Fugene HD (Promega), with a Fugene:DNA ratio of 3:1, for 24 hours. Plasmids harboring N-terminally myc-tagged wild-type or mutant human TDP-43 sequences (*TARDBP*, wild-type reference sequence NM_007375.3) were purchased from Origene. The presence of the A90V (c.269C>T) and M337V (c.1009A>G) mutations was confirmed by DNA sequencing performed by GENEWIZ (forward sequencing primer 5'-GGACTTTCCAAAATGTCG-3'; reverse sequencing primer 5’-ATTAGGACAAGGCTGGTGGG-3’). NLS-EGFP was a gift from Rob Parton (Addgene plasmid # 67652) [39].

In a subset of D-sorbitol stress experiments, HeLa cells were pre-treated with 50 μM of the irreversible pan-caspase inhibitor Z-Val-Ala-Asp-(OMe)-Fluoromethyl Ketone (Z-VAD-(OMe)-FMK, Cayman Chemical) or DMSO solvent control. After 30 min of caspase inhibition, 0.4 M of D-sorbitol or an equivalent volume of fresh medium was added and cells were incubated for a further 60 min before processing for immunofluorescence staining or fractionation of cells into enriched nuclear and cytosolic fractions.

To assess the rescue of TDP-43 and HuR cellular localization, cells were treated with 0.4 M D-sorbitol for 1 h and then allowed to recover in normal medium for 0 min, 15 min or 60 min.

### Protein biochemistry and Western blot analysis

For analysis of total protein, cells were lysed in radioimmunoprecipitation assay (RIPA) buffer (50 mM Tris HCl, 150 mM sodium chloride, 1% NP-40, 0.5% sodium deoxycholate, 0.1% sodium dodecyl sulfate [SDS]; Boston Bioproducts) supplemented with protease inhibitors (Complete, Roche), phosphatase inhibitors (PhosSTOP, Roche) and 2 mM ethylenediaminetetraacetic acid (EDTA). Cellular debris was removed by centrifugation at 16’000 x g for 15 min. Protein concentrations were determined using the bicinchoninic acid (BCA) assay (Thermo Fisher Scientific). Cellular fractionation experiments to obtain enriched cytosolic and nuclear fractions from cells grown on 60 mm dishes (Costar) were performed using a cell fractionation kit (Cell Signaling Technology) according to the manufacturer’s standard protocol. RIPA-soluble and–insoluble protein fractions were extracted according to a protocol adapted from Cohen *et al*. [40]. Briefly, following lysis of cells grown in a 6-well plate using 300 μl RIPA buffer supplemented with protease inhibitors (Complete, Roche) and 2 mM EDTA, lysates were sonicated twice for 15 seconds using a Soniprep 150 (MSE). Samples were then centrifuged at 100,000 x g and 4°C for 30 min in 1.5 ml microfuge tubes using an Optima TLX ultracentrifuge with a TLA100.4 rotor fitted with Delrin adaptors (all Beckman). The supernatant was collected as the RIPA-soluble fraction and the pellets were washed with 300 μl RIPA buffer and centrifuged at 100,000 x g and 4°C for 30 min. Pellets were extracted with 100 μl urea buffer (7 M urea, 2 M thiourea, 30 mM Tris pH 8.5, 4% 3-[(3-cholamidopropyl)dimethylammonio]-1-propanesulfonate (CHAPS)) and sonicated twice for 15 sec. Samples were centrifuged at 100,000 x g for 30 min at room temperature and the supernatant was collected as the RIPA-insoluble, urea-soluble fraction. For Western blots, both RIPA and urea fractions were diluted 1:1 with RIPA buffer.

Protein lysates and fractionated samples were separated on Tris-glycine SDS polyacrylamide gels (polyacrylamide concentrations ranging from 10–15%) and transferred onto polyvinylidene difluoride (PVDF, Millipore) membranes. Blocking solution and antibody dilutions were made up in 5% skimmed milk in Tris-buffered saline and 0.1% Tween-20 (TBS-T). Western blots were developed with enhanced chemiluminescent substrates (Clarity, BioRad; Visiglo HRP, Amresco). Digital images were acquired with a Chemidoc MP imaging system (BioRad). Where required, blots were stripped with stripping buffer for 15 min at room temperature (Thermo Fisher Scientific), blocked in 5% milk in TBS-T and reprobed with appropriate loading control antibodies.

### Immunofluorescence staining

Following stress treatments, cells were washed with phosphate-buffered saline (PBS) and fixed for 15 min with 4% paraformaldehyde (in PBS) before solubilization with 0.25% Triton-X (in PBS) for 10 min. Cells were washed again and blocked for 1 hour in 10% normal goat serum in PBS (Abcam). The fixed cells were incubated in primary antibody overnight at 4°C in a humidified chamber, and in secondary antibody for 1 hour at room temperature. Coverslips were mounted on microscope slides with Prolong Gold antifade with DAPI (Thermo Fisher Scientific) or counterstained with Hoechst 33342 (2 μg/ml; Thermo Fisher Scientific) and mounted with Prolong Gold antifade (Thermo Fisher Scientific). Cells transfected with NLS-EGFP were counterstained with Hoechst and mounted with Prolong Gold antifade. Images were acquired using a Nikon A1 confocal/Eclipse Ti inverted microscope system and NIS Elements software (Nikon).

### Antibodies

We used the following antibodies for detection of proteins by Western blot: rabbit carboxy- and amino-terminal TDP-43 (1:1,000–1:2,500; 12892-1-AP, 1:5,000; 10782-2-AP, Proteintech), mouse α-tubulin (1:15,000; ab80779, Abcam), mouse GAPDH (1:3,000; sc-32233, Santa Cruz), rabbit GAPDH (1:2,000; sc-25778, Santa Cruz), rabbit Lamin B1 (1:5,000; ab133741, Abcam), mouse histone H3 (1:6,000; 96C10, 3638, Cell Signaling Technology), rabbit caspase-3 (1:1,000; 8G10, 9665, Cell Signaling Technology), mouse caspase-9 (1:500; MAB8301, R&D Systems), mouse Nup153 QE5 (1:750; 906201, Biolegend). Anti-mouse and anti-rabbit horseradish peroxidase-coupled secondary antibodies were obtained from Jackson Immunoresearch (1:7,000). For immunofluorescence staining, we used rabbit carboxy- and amino-terminal TDP-43 (1:150 and 1:500), mouse myc 9E10 (1:200; sc-40, Santa Cruz) and mouse HuR (1:300; sc-5621, Santa Cruz). Secondary anti-mouse and anti-rabbit Alexa-488-, Alexa-555- and Alexa-568-coupled antibodies were purchased from Thermo Fisher Scientific.

### Quantification of immunofluorescence images and Western blots and statistical analysis

Western blot band densities were quantified with Image Lab 5 (BioRad). For immunofluorescence signal quantification of the cytosolic and nuclear TDP-43 ratios we used the Fiji open source program [41]. For statistical analysis, we used GraphPad Prism 6. For statistical analysis, we used either two-tailed Student’s t test or one-way ANOVA followed by Dunnett’s or Tukey’s multiple comparisons analysis as indicated for each experiment.

## Results

### Sorbitol triggers the formation of a 35 kDa truncated form of TDP-43 that is localized in the cytosol

**Fig 2:**
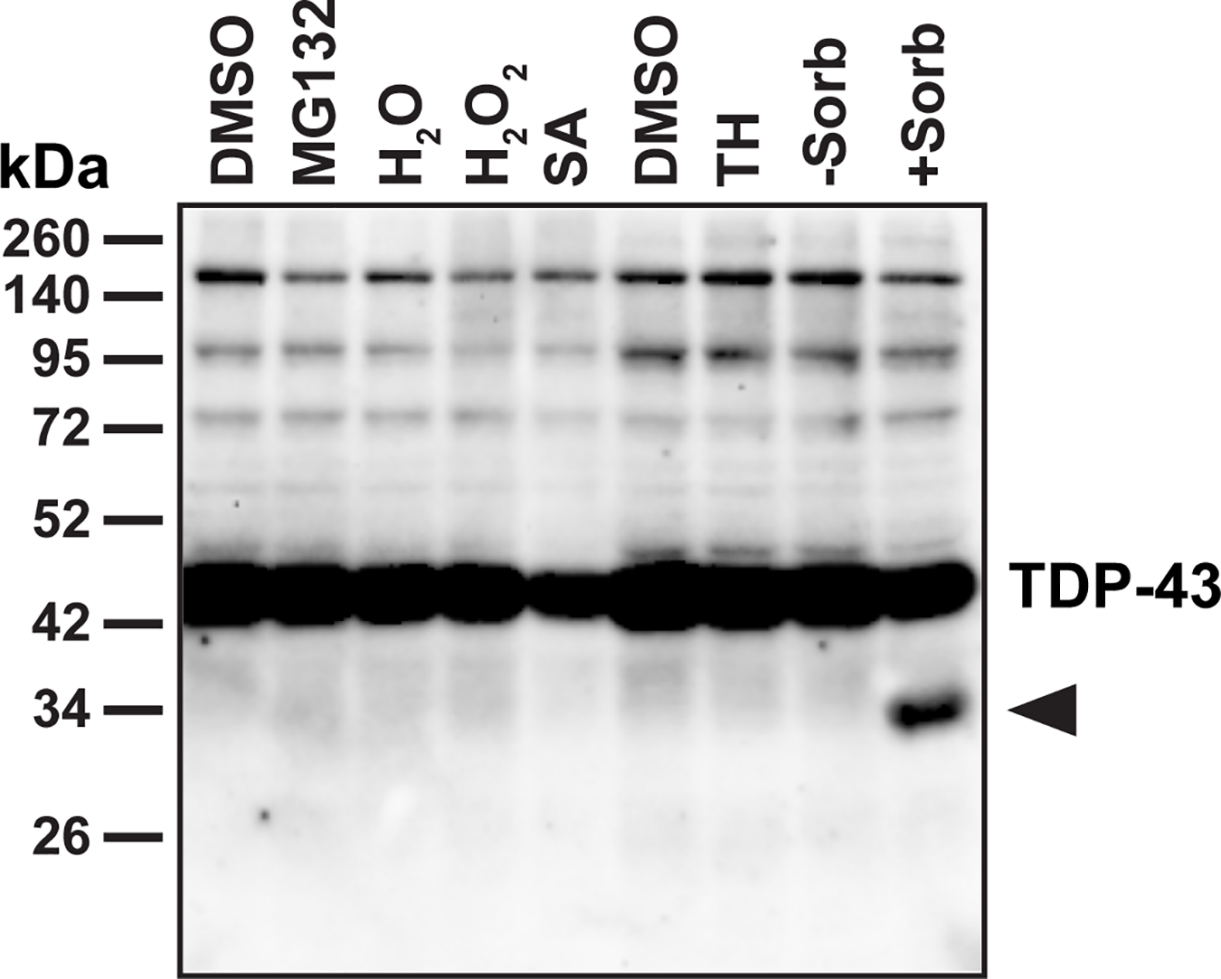
The osmotic and oxidative stressor D-sorbitol triggers cleavage of endogenous TDP-43 in HeLa cells. Untransfected HeLa cells expressing endogenous TDP-43 were treated with 10 μM MG132 for 4 h, 1 mM hydrogen peroxide (H_2_O_2_) for 1 h, 0.5 mM sodium arsenite (SA) for 30 min, 1 μM thapsigargin (TH) for 1 h, 0.4 M D-sorbitol (Sorb) for 1 h or with respective H_2_O and DMSO solvent controls. Western blot detection using a polyclonal TDP-43 antibody revealed a truncated product of TDP-43 with an approximate molecular weight of 35 kDa (CTF35, indicated by arrowhead) after treatment with D-sorbitol, but not with the other stressors.

First, we sought to identify a compound capable of inducing TDP-43 cleavage in a cellular system. To this end, we treated HeLa cells with various compounds described in the literature that were shown to induce pathological changes in TDP-43, though not necessarily TDP-43 truncation. We tested the proteasome inhibitor MG132 (10 μM, 4 h) [13, 42], the oxidative stressors hydrogen peroxide (1 mM, 60 min) [43] and sodium arsenite (0.5 mM, 30 min) [44], the sarco-endoplasmic reticulum Ca^2+^-ATPase inhibitor thapsigargin (1 μM, 60 min) [15, 29] and the oxidative and osmotic stressor D-sorbitol (0.4 M, 60 min) [14]. However, while MG132 as well as thapsigargin and D-sorbitol have been described to promote cleavage of TDP-43, we observed a reduction of full-length TDP-43 as well as the formation of a 35 kDa fragment (CTF35) with D-sorbitol only (Fig 2) [14, 29, 42]. We also detected HuR- and TDP-43-positive stress granules in a small number of sorbitol-treated cells (control: 0.57 ± 0.57% of cells; sorbitol: 11.4 ± 2.3% of cells, p = 0.017) (one-way ANOVA: F_2,6_ = 13.16, P = 0.0064, followed by Tukey’s multiple comparisons test) (S1 Fig). These findings are in line with Dewey *et al*., who showed that treatment of HEK293T cells with D-sorbitol leads to the association of TDP-43 with stress granules as well as to the formation of a 35 kDa cleavage product of TDP-43 [14]. In addition, we fractionated cell lysates of untreated or sorbitol-stressed HeLa cells into RIPA-soluble and RIPA-insoluble/urea-soluble proteins (S2 Fig). However, we did not find any evidence that sorbitol induces the aggregation of endogenous full-length TDP-43 or CTF35 into insoluble protein inclusions.

**Fig 3:**
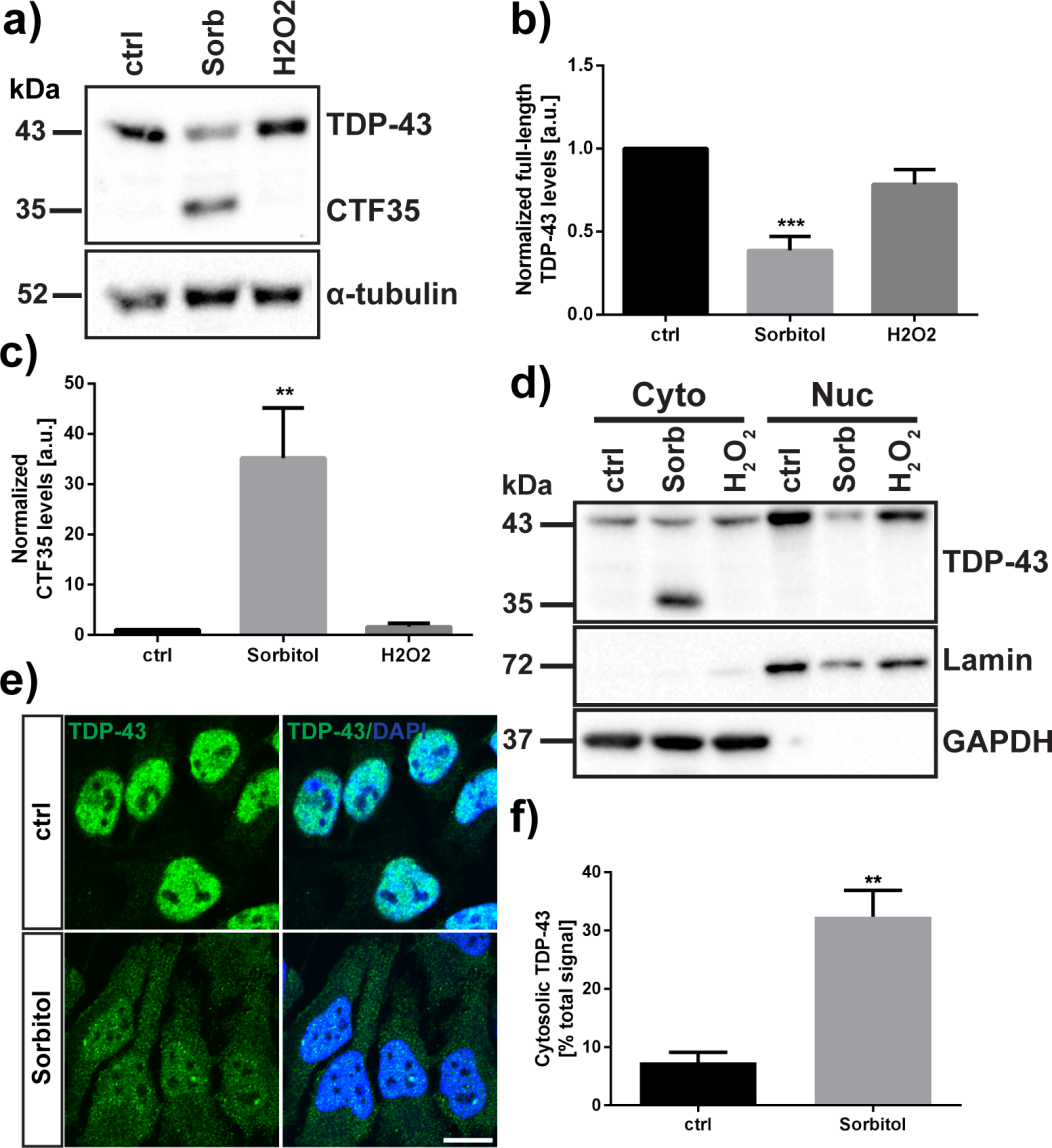
D-sorbitol induces the formation of a 35 kDa cleavage product of TDP-43 that is localized in the cytosol. (A) Western blot of TDP-43 protein (C-terminal antibody) after treatment of HeLa cells with 0.4 M D-sorbitol or 1 mM hydrogen peroxide for one hour. (B), (C) Densitometry analysis of (B) full-length TDP-43, (C) 35 kDa fragment of TDP-43 (CTF35). Densitometry results represent mean signal ± SEM (N = 4). (D) Detection of CTF35 in cytoplasmic (Cyto) and nuclear (Nuc) fractions of HeLa cells after treatment with D-sorbitol or hydrogen peroxide. (E) Immunofluorescence detection of cytoplasmic TDP-43 after D-sorbitol treatment. Scale bar = 15 μm. (F) Quantification of cytoplasmic TDP-43 immunofluorescence signal. Results represent mean TDP-43 immunofluorescence signal in the cytoplasm as a percentage of total TDP-43 signal in the cell ± SEM (N = 4). ** p < 0.01, *** p < 0.001.

We quantified both full-length TDP-43 and CTF35 in untreated, sorbitol- and hydrogen peroxide-treated HeLa cells and found that sorbitol led to a significant reduction in full-length TDP-43 (0.61 ± 0.08-fold decrease compared to control, p = 0.0003) (one-way ANOVA: F_2,9_ = 19.87, P = 0.0005, followed by Dunnett’s multiple comparisons test) and an increase in the 35 kDa cleavage product (34.1 ± 10.0-fold increase compared to control, p = 0.0044) (Fig 3A–3C). We observed no significant increase in the 35 kDa fragment after treatment with 1 mM hydrogen peroxide (0.67 ± 0.66-fold increase, p = 0.995) (one-way ANOVA: F_2,9_ = 11.41, P = 0.0034, followed by Dunnett’s multiple comparisons test).

**Fig 4:**
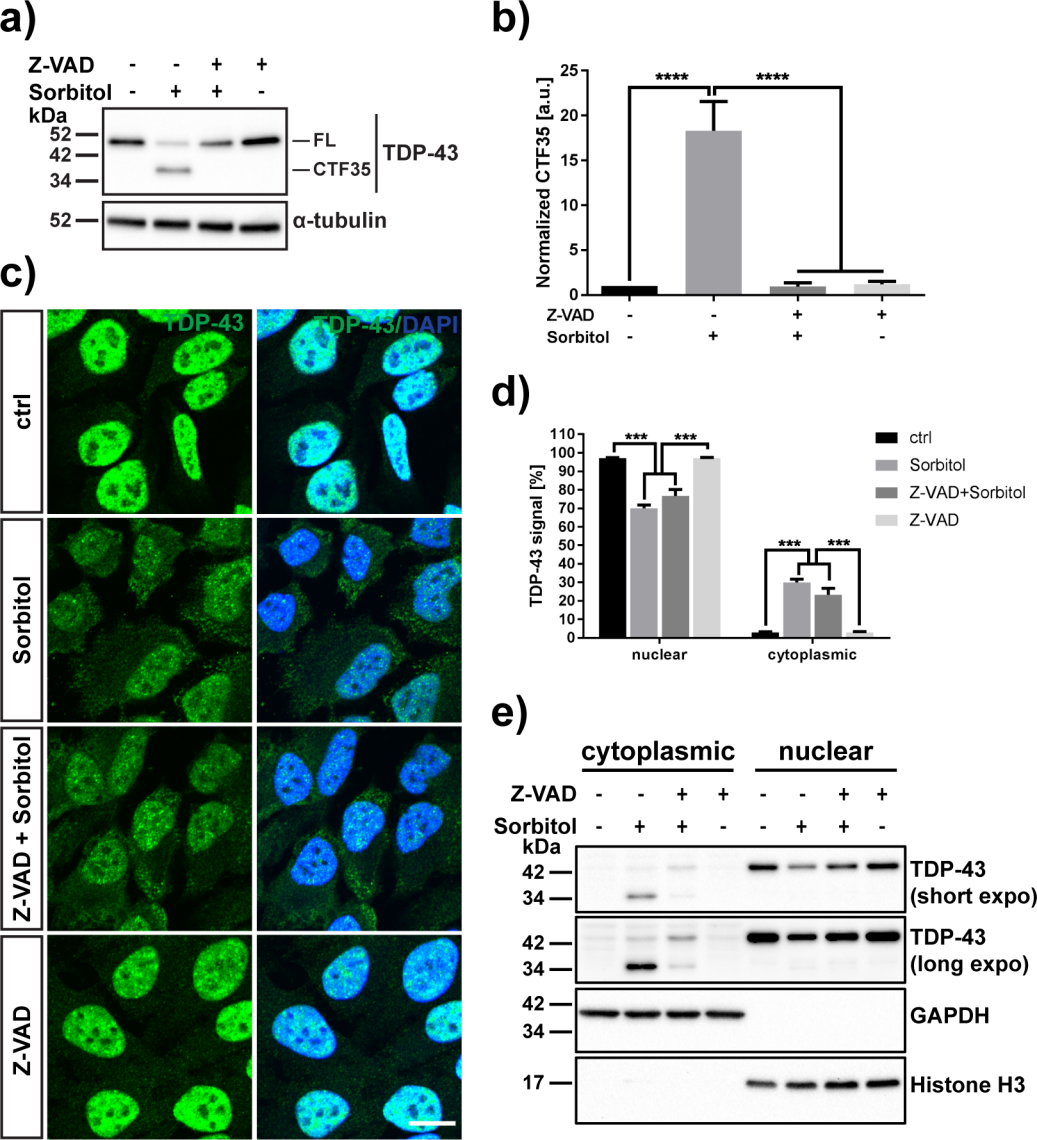
Inhibition of caspases prevents the formation of CTF35 of TDP-43, but does not prevent relocalization of the full-length protein to the cytosol. (A) TDP-43 Western blot of samples treated with or without D-sorbitol after pre-treatment with caspase inhibitor Z-VAD-FMK or DMSO control. α-tubulin was used as a loading control. FL = full-length TDP-43, CTF35 = 35 kDa C-terminal fragment of TDP-43. (B) Densitometry quantification of the 35 kDa fragment of TDP-43 after sorbitol stress in the absence or presence of caspase inhibitor (N = 5). (C) Immunofluorescence staining of nuclear and cytoplasmic TDP-43 using a C-terminal TDP-43 antibody. Cells were treated with or without D-sorbitol after pre-treatment with caspase inhibitor. Scale bar = 15 μm. (D) Quantification of nuclear and cytoplasmic TDP-43 fluorescence signals (N = 3). (E) Western blot of nuclear and cytoplasmic fractions of stressed HeLa cells. GAPDH was used as a control for the cytoplasmic fractions, histone H3 was chosen to confirm enrichment of the nuclear fraction. All results represent mean ± SEM; ** p < 0.01, *** p < 0.001.

By fractionating untreated and stressed HeLa cells into cytoplasmic and nuclear components, we detected CTF35 by Western blot in the cytoplasmic fraction in sorbitol-treated samples, while full-length TDP-43 in the nuclear fraction was reduced (Fig 3D, Fig 4C). Immunofluorescence staining of sorbitol-treated HeLa cells indicated that TDP-43 accumulated in the cytoplasm compared to control cells (control cells: 7.4 ± 1.7% of total TDP-43 signal; sorbitol-treated cells: 32.4 ± 4.5% of total TDP-43 signal; p = 0.0021, Student’s t test) (Fig 3E and 3F). While the antibody used for staining is unable to distinguish between full-length TDP-43 and the 35 kDa cleavage product, this result nevertheless supports the finding of a nuclear to cytoplasmic relocalization phenotype.

### Caspase inhibition blocks the sorbitol-induced formation of CTF35, but does not prevent the translocation of full-length TDP-43 to the cytoplasm

**Fig 5:**
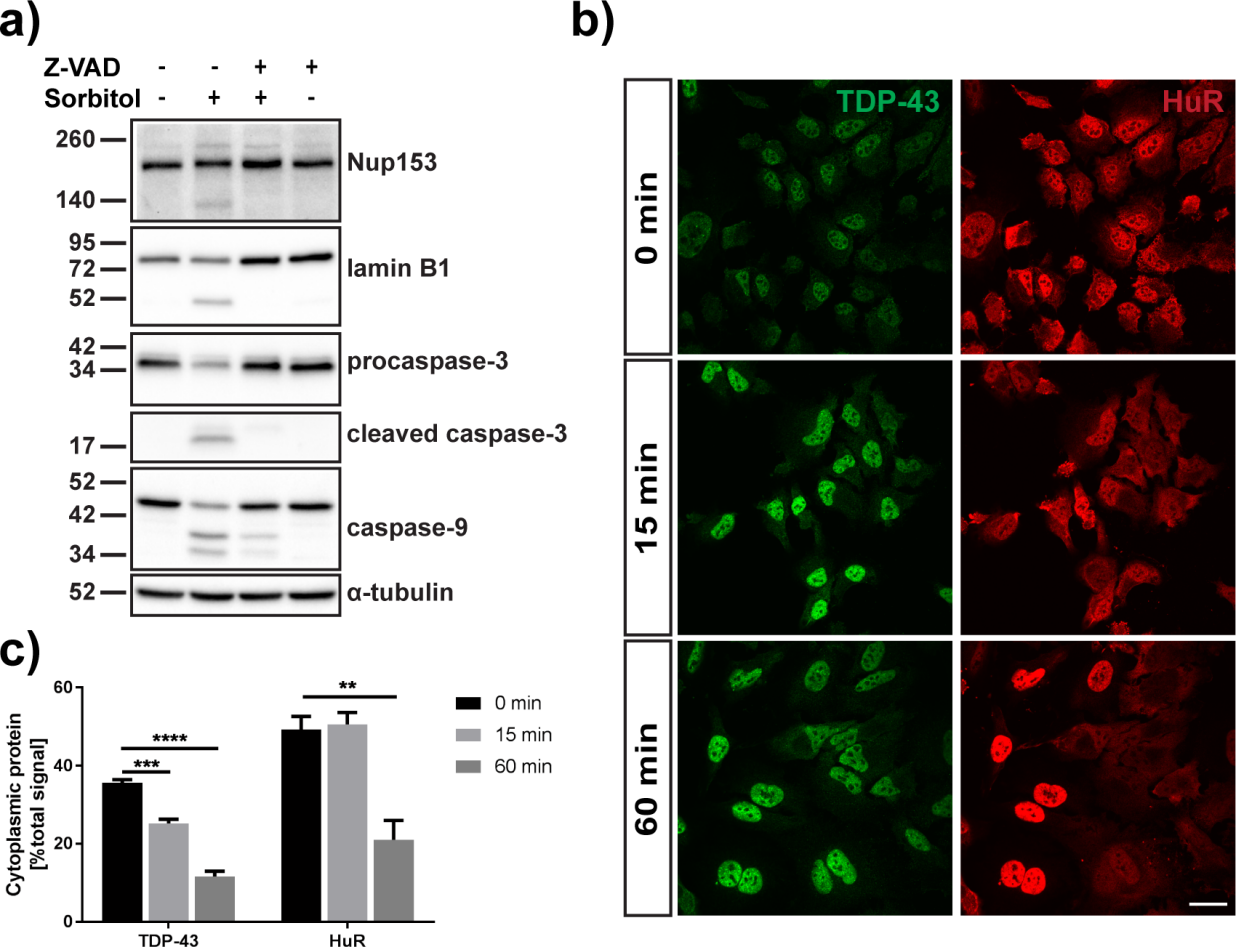
Sorbitol-dependent TDP-43 relocalization is reversible and not dependent on Nup153 cleavage. (A) Western blot of nuclear pore complex protein Nup153, nuclear lamina component lamin B1, procaspases -3 and -9 and their activated forms caspase-3 and caspase-9 in HeLa cells treated with sorbitol after pre-treatment with DMSO control or caspase inhibitor Z-VAD-FMK. α-tubulin was used as a loading control. (B) Immunofluorescence staining of nuclear-cytoplasmic shuttling proteins TDP-43 (green) and HuR (red) in sorbitol-stressed HeLa cells after 0 min, 15 min or 60 min rescue in normal medium. Scale bar = 30 μm. (C) Quantification of cytoplasmic TDP-43 and HuR protein in sorbitol-treated HeLa cells after 0 min, 15 min or 60 min rescue (N = 3). Results represent mean percentage of cytoplasmic signal ± SEM;** p < 0.01, *** p < 0.001, **** p < 0.0001.

In addition to TDP-43 cleavage, Dewey *et al*. also observed poly ADP ribose polymerase (PARP) cleavage in HEK293T cells treated with sorbitol, suggesting that these cells are undergoing apoptosis and thus caspase-3 is activated, which might in turn be responsible for the formation of the 35 kDa fragment of TDP-43 [14]. We wanted to confirm that the formation of CTF35 we detected in sorbitol-stressed HeLa cells was caused by activation of caspases. To this end, we pre-treated HeLa cells for 30 min with 50 μM of the irreversible pan-caspase inhibitor Z-VAD-(OMe)-FMK or DMSO control prior to adding 0.4 M sorbitol for one hour (Fig 4A and 4B). We found that pre-treatment of cells with the caspase inhibitor indeed abrogated the sorbitol-induced cleavage of TDP-43 and formation of CTF35 (Δ[sorb–Z-VAD/sorb] = 17.3 ± 3.3, p = 0.0001) (one-way ANOVA: F_3,16_ = 26.95, P < 0.0001, followed by Tukey’s multiple comparisons test), indicating that sorbitol induces the activation of caspases, which in turn cleave full-length TDP-43. Using a caspase-3-specific antibody, we confirmed that caspase-3 is activated by sorbitol, and that pre-treatment with ZVAD-FMK inhibits its activation (Fig 5A). Z-VAD-FMK also completely abrogated the cleavage of lamin B1, a known substrate of activated caspase-3 (Fig 5A) [45]. Furthermore, we showed that sorbitol-induced, caspase-mediated truncation of TDP-43 is a cell-type-dependent process, as treatment of SH-SY5Y cells with 0.4 M D-sorbitol for 1 hour neither led to activation of caspase-3 nor to the generation of CTF35 despite fulminant stress granule formation, indicating a functioning stress response (S3 Fig).

We then performed immunofluorescence staining of HeLa cells treated with sorbitol in the presence or absence of ZVAD-(OMe)-FMK in order to determine whether caspase inhibition can also rescue the TDP-43 relocalization from the nucleus to the cytoplasm (Fig 4C and 4D). However, we found no significant difference in cytoplasmic TDP-43 accumulation after sorbitol stress between untreated cells and cells treated with ZVAD-(OMe)-FMK in immunofluorescence staining with a carboxy-terminal TDP-43 antibody (cytoplasmic TDP-43: 42.5 ± 3.7% in cells treated with sorbitol, 38.5 ± 6.3% in cells treated with ZVAD + sorbitol; p = 0.864 (one-way ANOVA: F_3,8_ = 32.34, P < 0.0001, followed by Tukey’s multiple comparisons test) (Fig 4D). In order to confirm this result and to determine whether the cytoplasmic TDP-43 in samples treated with both caspase inhibitor and D-sorbitol was indeed full-length TDP-43, we fractionated untreated and treated cell samples into nuclear and cytoplasmic fractions and resolved them by SDS-PAGE (Fig 4E). While we found, as expected, that pre-treatment with Z-VAD-FMK abrogated the sorbitol-induced formation and cytoplasmic accumulation of CTF35, we detected an accumulation of full-length, uncleaved TDP-43 protein in the cytoplasmic fraction, suggesting that TDP-43 cleavage is not a prerequisite for trapping the protein in the cytoplasm. Caspase inhibition also did not rescue the formation of TDP-43- and HuR-positive stress granules in response to sorbitol (sorbitol: 11.4 ± 2.3% of cells; Z-VAD + sorbitol: 13.6 ± 2.3% of cells, p = 0.71) (one-way ANOVA: F_2,6_ = 13.16, P = 0.0064, followed by Tukey’s multiple comparisons test) (S1 Fig).

### Sorbitol-induced cytoplasmic relocalization is a reversible phenomenon not limited to TDP-43

The nuclear envelope is the cellular barrier that demarcates the nucleus. During apoptosis, nuclear permeability increases due to the breakdown of the nuclear pore complex (NPC), which regulates traffic of large macromolecules between the cytosol and nucleus [46–48]. As a result, proteins found in the nucleus disperse throughout the cytoplasm, while cytoplasmic proteins enter the nucleus. Nup153, a component of the NPC thought to be crucially involved in modulating nuclear permeability, has been shown to be cleaved by caspases [49, 50]. We found that sorbitol indeed initiates cleavage of Nup153; however, the majority of Nup153 remained uncleaved after one hour of sorbitol treatment (Fig 5A). Furthermore, pre-treatment of HeLa cells with the caspase inhibitor Z-VAD-FMK abrogated the cleavage of Nup153. We thus conclude that the cytoplasmic relocalization of TDP-43 as a response to sorbitol stress is not the result of degradation of Nup153, as TDP-43 accumulates in the cytoplasm in HeLa cells pre-treated with caspase inhibitor prior to the sorbitol insult. We also investigated the activity of caspase-9, an initiator caspase that modulates nuclear leakiness by affecting hydrophobic proteins which form a permeability barrier in the NPC through hydrophobic protein-protein interactions [46, 51]. We detected cleaved caspase-9, an indicator of autocatalytic activity, in HeLa cells treated with sorbitol (Fig 5A). While pre-treatment with the pan-caspase inhibitor Z-VAD-FMK partially suppressed caspase-9 autocatalytic cleavage, we still observed some caspase-9 activity. As activation of caspase-9 has been shown to be required for the release of GFP fused to a nuclear localization signal (NLS-GFP) [46], we sought to determine whether sorbitol affects the localization of this fusion protein. To this end, we treated HeLa cells transfected with NLS-EGFP with sorbitol for one hour. In control cells, we detected NLS-EGFP predominantly in the nuclear compartment, while in sorbitol-treated cells, the percentage of nuclear GFP signal was significantly lower (control: 73.2 ± 1.5% of total EGFP signal; sorbitol: 62.8 ± 3.3% of total EGFP signal, p = 0.041) (S4 Fig). Pre-treatment with Z-VAD-FMK did not rescue the relocalization of NLS-EGFP (Z-VAD + sorbitol: 58.1 ± 3.2% of total EGFP signal, p = 0.010) (one-way ANOVA: F_2,8_ = 7.754, P = 0.0134, followed by Dunnett’s multiple comparisons test).

Finally, we performed a rescue experiment to determine whether the changes in nuclear permeability and TDP-43 mislocalization caused by the sorbitol insult were reversible. Following a one hour exposure to 0.4 M D-sorbitol, cells were left to recover in fresh normal medium for 0 min, 15 min or 60 min (Fig 5B and 5C). In recovering cells, TDP-43 in the cytoplasm (as a percentage of total TDP-43 signal) gradually decreased, with cytoplasmic TDP-43 35.6 ± 0.8%, 25.6 ± 1.0% and 11.6 ± 1.4% of total TDP-43 after 0 min, 15 min and 60 min of recovery, respectively (one-way ANOVA: F_2,6_ = 119.7, P < 0.0001, followed by Dunnett’s multiple comparisons test: 0 vs. 15 min p = 0.001, 0 vs. 60 min p = 0.001). This decrease in the cytoplasmic-to-nuclear TDP-43 ratio was paralleled by an increase in nuclear TDP-43 signal (S5A Fig). This result indicates that the nuclear changes leading to the mislocalization of TDP-43 in response to sorbitol stress are transient and reversible. We also investigated the rescue of HuR localization, another nuclear-cytoplasmic shuttling, RNA-binding protein (Fig 5B and 5C). While the cytoplasmic-to-nuclear HuR signal ratio (0 min: 50.6 ± 3.1%; 60 min: 21.0 ± 4.9%) as well as total nuclear HuR significantly recovered after a 60 minute rescue period, we observed no improvement after 15 min (0 min: 50.6 ± 3.1%; 15 min: 50.6 ± 3.1%) (one-way ANOVA: F_2,6_ = 18.65, P = 0.0027, followed by Dunnett’s multiple comparisons test: 0 vs. 15 min p = 0.96, 0 vs. 60 min p = 0.0037) (Fig 5C, S5B Fig). Furthermore, while the nuclear-cytoplasmic distribution of TDP-43 appeared homogenous between cells after 15 and 60 min of recovery, we observed heterogeneous cell populations in the HuR staining, with some cells showing a clear nuclear localization of HuR, while others–despite nuclear TDP-43 localization–displayed an even distribution of HuR throughout the cell.

### The A90V variant of TDP-43 confers resistance against caspase-induced cleavage

**Fig 6:**
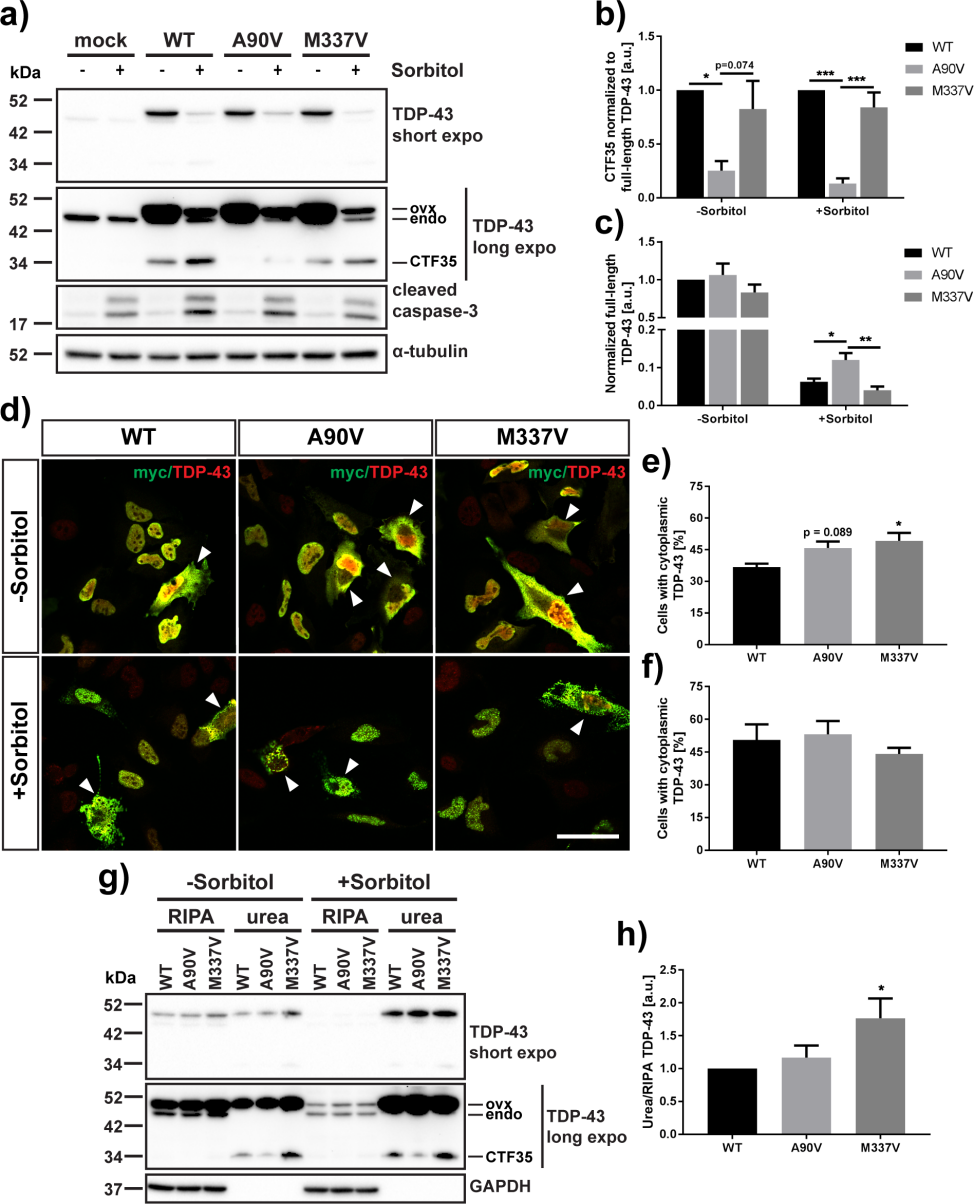
The A90V mutation confers resistance against caspase-mediated cleavage of TDP-43 within the nuclear localization sequence. (A) Western blot detection of full-length and 35 kDa fragment of TDP-43 as well as cleaved caspase-3 in HeLa cells transfected with WT, A90V or M337V mutant TDP-43 and treated with 0.4 M D-sorbitol for 1 hour. ovx = overexpressed WT, A90V or M337V TDP-43, endo = endogenous TDP-43. α-tubulin was used as a loading control. (B), (C) Western blot quantification of (B) cleaved 35 kDa TDP-43 normalized to full-length TDP-43, (C) full-length TDP-43 in untreated HeLa cells and cells after treatment with D-sorbitol. Result represents mean signal ± SEM (N = 4). (D) Immunofluorescence staining of control or sorbitol-stressed HeLa cells transfected with WT, A90V or M337V TDP-43 and probed with anti-myc (green) and anti-TDP-43 (red) antibodies. Cells with cytoplasmic TDP-43 are indicated by arrowhead. Scale bar = 50 μm. (E), (F) Quantification of transfected cells with cytoplasmic TDP-43 under control (E) or sorbitol (F) conditions. Result represents mean percentage of transfected cells with cytoplasmic TDP-43 ± SEM (N = 5). (G) Western blot detection of RIPA-soluble and urea-soluble TDP-43 WT, A90V or M337V in untreated and sorbitol-treated HeLa cells. GAPDH was used as a fractionation control. (H) Quantification of total TDP-43 (full-length + CTF35) in RIPA fraction normalized to TDP-43 in urea fraction in untreated cells. Results represent mean normalized urea/RIPA ratio ± SEM (N = 6). * p < 0.05, ** p < 0.01, *** p < 0.001, one-way ANOVA followed by Tukey’s or Dunnett’s multiple comparisons test.

The caspase cleavage site resulting in the formation of the TDP-43 cleavage product CTF35 lies after aspartic acid 89 (Fig 1). Introduction of an aspartic acid to glutamic acid mutation at this amino acid (D89E) confers resistance against caspase-mediated cleavage [27, 29, 32]. This mutation has not been described in any ALS or FTD patients, and has only been used as a proof of concept to confirm the actual caspase cleavage site. However, an alanine to valine amino acid substitution at residue 90 (A90V) has been described in an FTLD/ALS patient with a family history of dementia [52]. Using D-sorbitol as an inducer of caspase activity, we sought to determine whether the A90V mutation, which lies directly next to the carboxy-side of the second aspartic acid of the caspase-3 recognition motif within the NLS, has an effect on TDP-43 cleavage propensity at that site (Fig 6A and 6B). After 24 h of transfection, we detected low levels of CTF35 in HeLa cells even in the absence of sorbitol stress (Fig 6A). Following exposure of transfected cells with D-sorbitol, we saw an increase in the cleaved fragment. Interestingly, less CTF35 was detected in A90V-transfected cells compared to WT and M337V mutant TDP-43 both in untreated (Δ[WT-A90V]: 0.748 ± 0.090, p = 0.0224; Δ[M337V-A90V]: 0.573 ± 0.277, p = 0.074) (one-way ANOVA: F_2,9_ = 5.989, P = 0.022, followed by Tukey’s multiple comparisons test) and sorbitol-treated cells (Δ[WT-A90V]: 0.868 ± 0.049, p = 0.0001; Δ[M337V-A90V]: 0.708 ± 0.148, p = 0.0006) (one-way ANOVA: F_2,9_ = 29.3, P = 0.0001, followed by Tukey’s multiple comparisons test) (Fig 6B). Furthermore, there was significantly more full-length TDP-43 present in A90V-transfected cells compared to WT and M337V-transfected cells following D-sorbitol treatment (Δ[WT-A90V]: 0.058 ± 0.021, p = 0.032; Δ[M337V-A90V]: 0.080 ± 0.021, p = 0.005), suggesting that full-length TDP-43 is partially rescued from cleavage or degradation (one-way ANOVA: F_2,9_ = 9.77, P = 0.0056, followed by Tukey’s multiple comparisons test) (Fig 6C). As we detected robust activation of caspase-3 following treatment with D-sorbitol regardless of the expressed TDP-43 construct, the lower levels of CTF35 in A90V-expressing cells were not due to a lack of caspase activity (Fig 6A). We thus conclude that the alanine to valine amino acid substitution at residue 90 confers a resistance against caspase-mediated cleavage at that site.

We also investigated the subcellular localization of WT and mutant TDP-43 in HeLa cells (Fig 6D–6F). We found a significant increase in cells with cytoplasmic TDP-43 in M337V mutant cells compared to WT-expressing cells (WT: 36.8 ± 1.6%, M337V: 49.2 ± 3.7% of transfected cells; p = 0.021) (Fig 6D and 6E). Similarly, we found a trend towards an increase in the number of A90V-expressing cells with cytoplasmic TDP-43 compared to WT (A90V: 45.9 ± 3.0% of transfected cells, p = 0.089) (one-way ANOVA: F_2,12_ = 4.758, P = 0.03, followed by Dunnett’s multiple comparisons test). These results are in line with published literature showing cytoplasmic accumulation of TDP-43 in A90V- and M337V-expressing cells *in vitro* [52, 53]. In contrast, we did not find a significant difference in the proportion of WT-, A90V- or M337V-transfected cells with cytoplasmic TDP-43 after a one hour treatment with sorbitol (WT: 50.5 ± 7.2%, A90V: 53.1 ± 6.1%, M337V: 44.2 ± 2.8% of transfected cells) (one-way ANOVA followed by Dunnett’s multiple comparisons test, F_2,12_ = 0.664, P = 0.533) (Fig 6E and 6F). However, in sorbitol-treated cells the overexpressed myc-TDP-43 displayed a granular staining pattern, which could suggest the formation of insoluble, aggregated TDP-43 (S6 Fig). To confirm this, we fractionated lysates of control and sorbitol-treated HeLa cells into RIPA-soluble and RIPA-insoluble, urea-soluble material (Fig 6G and 6H). We detected both full-length TDP-43 and CTF35 in the urea fraction in untreated HeLa cells. We only observed very low levels of CTF35 in the RIPA-soluble fraction. When we quantified TDP-43 in the urea fraction (full-length + CTF35, normalized to TDP-43 in the RIPA-soluble fraction) in untreated cells, we found significantly more TDP-43 in M337V-mutant expressing cells compared to WT (Δ = 76.5 ± 30.3%, p = 0.0334) (Fig 6H). There was no significant increase in insoluble TDP-43 in A90V-expressing cells (Δ = 16.8 ± 18.4%, p = 0.787) (one-way ANOVA: F_2,15_ = 3.873, P = 0.0441, followed by Dunnett’s multiple comparisons test). Sorbitol treatment caused a decrease in RIPA-soluble TDP-43 and an increase in TDP-43 in the urea fraction, confirming that sorbitol promotes the aggregation of overexpressed TDP-43 regardless of mutation. Last, we investigated stress granule formation in HeLa cells overexpressing TDP-43. We detected TDP-43-positive stress granules in a very small number of untreated cells (<4% of transfected cells). Sorbitol treatment caused an increase in the number of cells with stress granules (>8% of transfected cells). However, we found no difference in the number of cells with TDP-43-positive stress granules between genotypes under either control or stress conditions (S7 Fig). However, this finding does not exclude the possibility that the A90V and/or M337V mutations have more subtle effects on stress granule composition or dynamics.

## Discussion

While the occurrence of C-terminal fragments of TDP-43 is a common pathological hallmark in ALS and FTD pathology, the significance of TDP-43 truncation for disease pathogenesis remains unclear. It is unknown whether TDP-43 fragments are disease-relevant species or a by-product of the cellular protein degradation machineries’ attempt to get rid of toxic full-length TDP-43 species. For example, whether C-terminal fragments affect cell viability is uncertain, with various papers suggesting reduced [29], similar [22] or increased [31] toxicity compared to full-length TDP-43 depending on the fragment used and expression system chosen. On the other hand, the current literature suggests that TDP-43 CTFs are prone to aggregation and hyperphosphorylation, and as they lack a nuclear localization sequence, accumulate in the cytoplasm [22, 28, 54, 55]. These features are defining hallmarks of TDP-43 proteinopathies.

In the current study, we have adapted a protocol by Dewey *et al*. that describes the use of high concentrations of D-sorbitol, a sugar alcohol, as an osmotic and oxidative stressor in HEK293T cells that drives TDP-43 cleavage, resulting in the generation of a 35 kDa fragment [14]. D-sorbitol use as a stressor to elicit TDP-43 cleavage is fast-acting, with the formation of the CTF35 TDP-43 fragment occurring within one hour of treatment in HeLa cells in our experiments. In a first effort, we sought to confirm whether the sorbitol-induced cleavage of TDP-43 and the formation of the CTF35 fragment are indeed mediated by the activation of caspases. Dewey *et al*. showed that cleavage of Poly(ADP-ribose) polymerase (PARP), a known substrate of caspase-3, preceded cleavage of TDP-43 in sorbitol-treated HEK293T cells [14]. However, this finding is not a direct proof, as caspase-independent PARP cleavage has previously been described [56, 57]. By pre-treating HeLa cells with the pan-caspase inhibitor Z-VAD-FMK prior to sorbitol stress, we abrogated TDP-43 cleavage, confirming that the formation of the 35 kDa TDP-43 fragment CTF35 in response to sorbitol stress is indeed caspase-dependent, as suggested by Dewey *et al*. This is in line with the literature showing that CTF35 is formed in response to caspase activation [29, 32].

By fractionating cells into nuclear and cytoplasmic fractions, we showed that CTF35 accumulates in the cytoplasm of sorbitol-treated HeLa cells, while full-length protein in the nucleus is depleted. However, while pre-treatment of cells with caspase inhibitor prevented the cleavage of TDP-43, we found that it did not rescue the relocalization of full-length TDP-43 from the nucleus to the cytoplasm. This result suggests that C-terminal protein truncation is not necessary to trap TDP-43 in the cytoplasm and that the cytoplasmic relocalization of TDP-43 likely precedes its caspase-dependent cleavage. We also found that HuR, another nuclear-cytoplasmic shuttling protein, relocalizes to the cytoplasm in response to sorbitol, as does NLS-EGFP, albeit with a smaller effect size than seen with TDP-43 and HuR. The accumulation of TDP-43 in the cytoplasm is thus likely the result of sorbitol-induced leakiness of the nuclear envelope. One of the factors known to regulate nuclear permeability is the nuclear pore complex component Nup153, which is degraded in apoptotic cells [46, 48, 49]. We only found a small fraction of Nup153 cleaved in response to sorbitol, and cleavage was completely suppressed by caspase inhibition, indicating that the degradation of this NPC protein is not responsible for the observed nuclear leakiness. Furthermore, Z-VAD-FMK did not completely inhibit the autocatalytic cleavage of caspase-9, an initiator caspase that regulates nuclear transport and increases nuclear leakiness by perturbing the permeability barrier inside the NPC [46, 51]. The possibility that sorbitol-induced redistribution of TDP-43 is dependent on caspase-9 is in line with a study showing that the expression of a dominant-negative form of caspase-9 prevents nuclear leakiness and the collapse of the Ran gradient required for nuclear import [46]. Furthermore, several studies have shown that oxidative and osmotic stressors can trigger the collapse of the Ran gradient [58, 59]. Interestingly, elevated serum levels of caspase-9 as well as activated caspase-9 in spinal cord have been described in ALS patients [60, 61]. We also demonstrated that the sorbitol-induced changes in nuclear permeability are transient, as TDP-43 and–with a lag time–HuR relocalized to the nucleus after a recovery period. In conclusion, we have demonstrated that the sorbitol-induced, reversible redistribution of TDP-43 is the result of either caspase-independent or caspase-9-dependent changes in nuclear permeability and/or nuclear transport machinery that precede the breakdown of the nuclear envelope.

We also used sorbitol to investigate the cleavage propensity and cytoplasmic accumulation of the A90V variant of TDP-43. This variant lies within the bipartite nuclear localization sequence of TDP-43 directly adjacent to the caspase-3 cleavage site after Asp89. Genetic studies have detected the A90V variant both in ALS cases as well as in healthy control individuals, leaving the impact of this single amino acid mutation on disease pathogenesis unclear [52, 62–64]. A recent *in vitro* study has shown that A90V-TDP-43 protein incubated with purified caspase-3 is less efficiently cleaved than WT-TDP-43 [65]. We addressed the question of the impact of the A90V sequence variant on TDP-43 cleavage using a cellular system, by transfecting HeLa cells with WT, A90V or M337V TDP-43 before treatment with D-sorbitol. We found that even in the absence of sorbitol, CTF35 is formed and found predominantly aggregated, and that it accumulates further after sorbitol stress. Both with and without sorbitol treatment, the A90V variant was less efficiently cleaved at the Asp89 residue than WT or M337V TDP-43. Interestingly, recent studies have shown that the cleavage-resistant D89E TDP-43 mutant confers no toxicity in a cellular expression system, while the disease-related D169G mutation, which is more efficiently cleaved at Asp89 than WT TDP-43, is highly toxic [27, 65]. This suggests that CTF35 might be an important mediator of cellular toxicity. On the other hand, we found a trend towards increased cytoplasmic TDP-43 in the A90V mutant in line with published literature [52, 66]. This apparent dichotomy between increased cytoplasmic accumulation and decreased generation of toxic fragments could explain why this variant is found both in patients and healthy controls, as the balance between increased toxicity due to TDP-43 mislocalization and reduced toxicity due to reduced formation of C-terminal fragments might be tipped in each direction depending on the carriers’ specific genetic makeup and environmental exposure to additional risk factors.

In summary, we have described here the use of D-sorbitol as an inducer of caspase-dependent cleavage of TDP-43 to investigate the relationship between TDP-43 cleavage and cytoplasmic relocalization. We also looked at the impact of these phenotypes in the context of the TDP-43 mutants A90V and M337V and observed cleavage resistance of the A90V variant in a cellular context.

At the time this study was conducted, the cleavage resistance of the A90V variant at the Asp89 cleavage site was not published. However, a recent paper published by Chiang et al. demonstrated this resistance against caspase-3-mediated cleavage *in vitro* [65].

## Supporting information

S1 Fig Sorbitol induces the formation of stress granules in HeLa cells. HeLa cells were either pre-treated with pan-caspase inhibitor Z-VAD-FMK or DMSO control before 0.4 M D-sorbitol was added as a stressor for 1 h. Control cells were not treated with either sorbitol or Z-VAD-FMK. (A) Example of immunofluorescence staining of untreated and sorbitol-stressed HeLa cells. Stress granules (indicated by arrowhead) are defined as cytoplasmic puncta co-stained with TDP-43 (green) and the stress granule marker HuR (red). Scale bar = 30 μM. (B) Quantification of cells with a minimum of three TDP-43^+^/HuR^+^ stress granules (N = 4). Results represent mean number of cells with stress granules ± S.E.M.; * p < 0.05, ** p < 0.01; one-way ANOVA (F_2,6_ = 13.16, P = 0.0064) followed by Tukey’s multiple comparisons test.
(TIF)

S2 Fig Sorbitol does not cause the formation of insoluble endogenous full-length TDP-43 or its 35 kDa cleavage fragment. HeLa cells were treated with 1 mM H_2_O_2_, 1 μM thapsigarin (TH) or 0.4 M D-sorbitol. Full-length TDP-43 and its 35 kDa cleavage product (marked with asterisk) were detected in the RIPA-soluble protein fraction (R), but not in the RIPA-insoluble, urea-soluble fraction (U). Arrowhead denotes unspecific band.
(TIF)

S3 Fig Sorbitol causes caspase-3 activation and TDP-43 cleavage in HeLa, but not in SH-SY5Y cells. A) HeLa cells and undifferentiated SH-SY5Y cells were treated with 0.5 mM (SH-SY5Y) or 1 mM (HeLa) hydrogen peroxide (H_2_O_2_), 1 μM thapsigargin (TH) or 0.4 M D-sorbitol. The generation of CTF35 as well as the activation of caspase-3 was observed in HeLa cells after 1 hour of D-sorbitol treatment, but not in SH-SY5Y cells. B) Prominent formation of HuR-positive stress granules in SH-SY5Y cells in response to sorbitol (indicated with arrowhead). Blue = DAPI, green = TDP-43, red = HuR. Scale bar = 30 μm.
(TIF)

S4 Fig Sorbitol causes the cytoplasmic accumulation of NLS-EGFP. (A) Immunofluorescence images of HeLa cells transfected with EGFP fused to a nuclear localization sequence. After 24 h, cells were treated with DMSO or Z-VAD-FMK for 30 min, followed by regular medium or 0.4 M sorbitol for 60 min. Scale bar = 20 μm. (B) Quantification of nuclear NLS-EGFP signal as a proportion of total cellular EGFP signal (N = 3–4). Results represent mean percentage of nuclear GFP signal ± S.E.M.; * p < 0.05; one-way ANOVA (F_2,8_ = 7.754, P = 0.0134) followed by Dunnett’s multiple comparisons test.
(TIF)

S5 Fig Increase in nuclear TDP-43 and HuR after cessation of sorbitol stress. HeLa cells were exposed to 0.4 M D-sorbitol for 1 h, then left to recover for 0 min, 15 min or 60 min. (A) Nuclear TDP-43 and (B) HuR signals were quantified. Results represent mean nuclear immunofluorescence per cell ± SEM; ** p < 0.01 (N = 3); one-way ANOVA (TDP-43: F_2,6_ = 10.34, P = 0.0114; HuR: F_2,6_ = 18.72, P = 0.0026) followed by Dunnett’s multiple comparisons test.
(TIF)

S6 Fig myc-TDP-43 forms granular pattern under sorbitol stress conditions. TDP-43 was expressed in HeLa cells for 24 h before sorbitol was added for 1 h. Overexpressed myc-TDP-43 forms a granular staining pattern in the nucleus indicative of protein aggregation both in cells with (blue rectangle) and without (orange rectangle) cytoplasmic TDP-43 relocalization (WT-transfected example).
(TIF)

S7 Fig The A90V variant does not inhibit stress granule formation. HeLa cells were transfected with WT, A90V or M337V TDP-43. After 24 h, cells were either left untreated or were treated with 0.4 M sorbitol for 1 h. (A) Immunofluorescence staining of transfected HeLa cells with or without additional sorbitol treatment. Green = myc, red = TDP-43. Arrowheads indicate stress granules. (B), (C) Quantification of proportion of transfected cells with TDP-43-positive stress granules in control conditions (B) or after sorbitol stress (C). Results represent mean percentage of transfected cells with TDP-43-positive cytoplasmic granules ± SEM. one-way ANOVA followed by Tukey’s multiple comparisons test (-Sorbitol: F_2,12_ = 1.098, P = 0.365; +Sorbitol: F_2,12_ = 0.3245, P = 0.729).
(TIF)
