# Supplementary figures and images for: Truncation of the TAR DNA-binding protein 43 is not a prerequisite for cytoplasmic relocalization, and is suppressed by caspase inhibition and by introduction of the A90V sequence variant
Source: PLoS One. 2017 May 16;12(5):e0177181. doi: 10.1371/journal.pone.0177181 (PMC5433705; doi:10.1371/journal.pone.0177181)

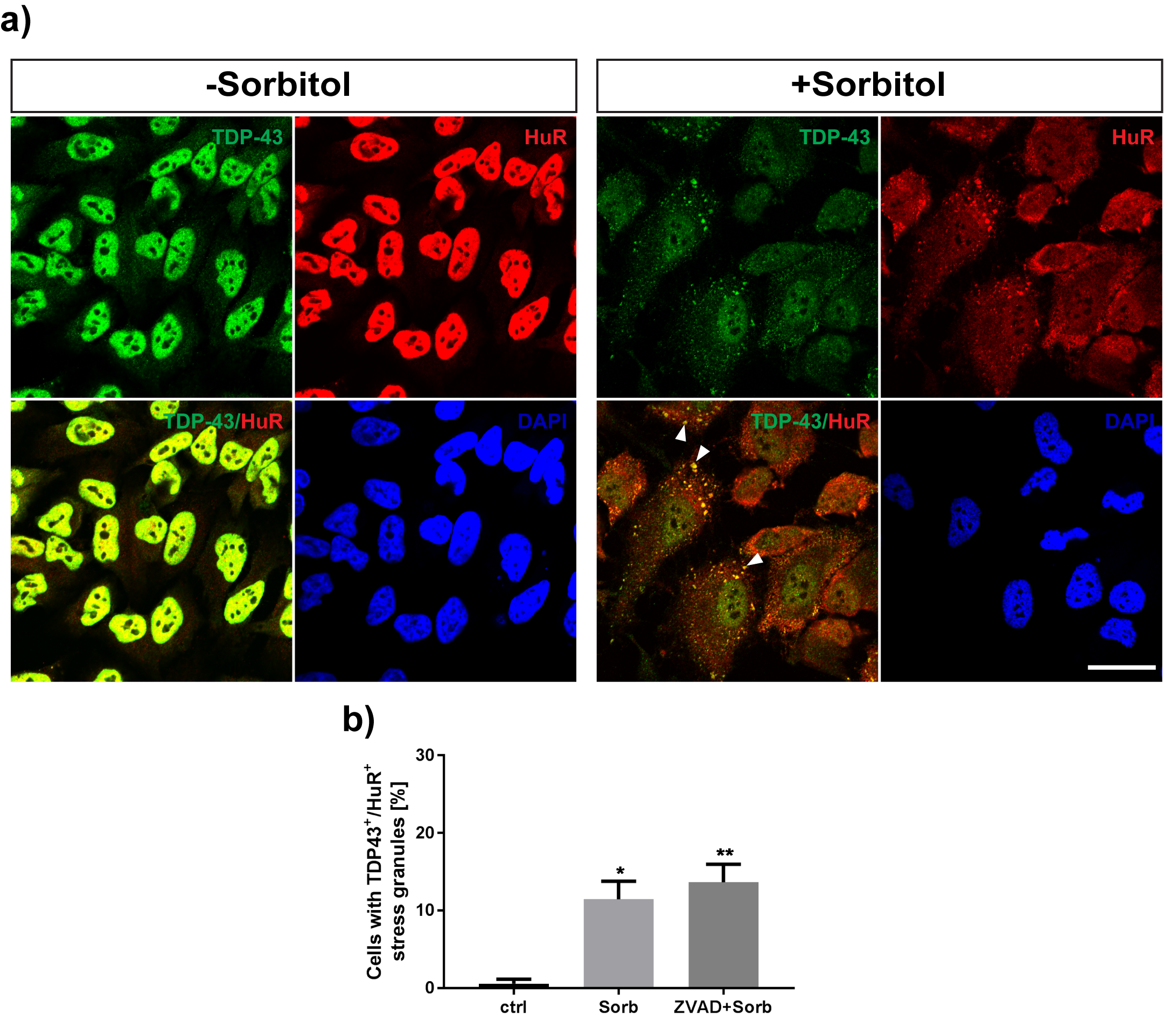

Supplement: S1 Fig — HeLa cells were either pre-treated with pan-caspase inhibitor Z-VAD-FMK or DMSO control before 0.4 M D-sorbitol was added as a stressor for 1 h. Control cells were not treated with either sorbitol or Z-VAD-FMK. (A) Example of immunofluorescence staining of untreated and sorbitol-stressed HeLa cells. Stress granules (indicated by arrowhead) are defined as cytoplasmic puncta co-stained with TDP-43 (green) and the stress granule marker HuR (red). Scale bar = 30 μM. (B) Quantification of cells with a minimum of three TDP-43+/HuR+ stress granules (N = 4). Results represent mean number of cells with stress granules ± S.E.M.; * p < 0.05, ** p < 0.01; one-way ANOVA (F2,6 = 13.16, P = 0.0064) followed by Tukey’s multiple comparisons test. (TIF) [file pone.0177181.s001.tif]

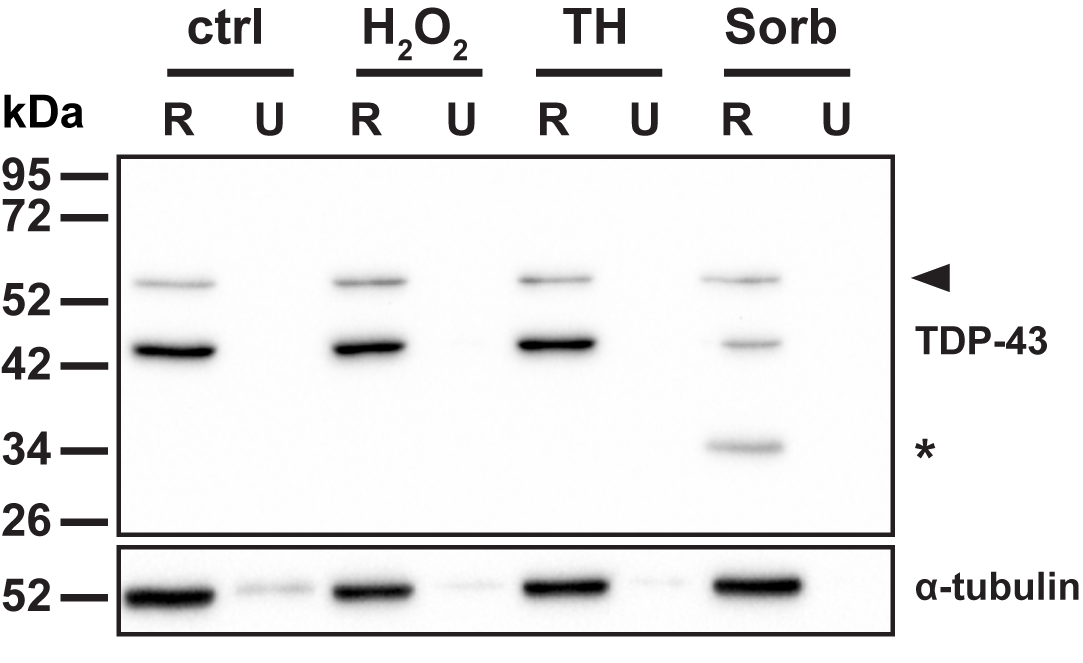

Supplement: S2 Fig — HeLa cells were treated with 1 mM H2O2, 1 μM thapsigarin (TH) or 0.4 M D-sorbitol. Full-length TDP-43 and its 35 kDa cleavage product (marked with asterisk) were detected in the RIPA-soluble protein fraction (R), but not in the RIPA-insoluble, urea-soluble fraction (U). Arrowhead denotes unspecific band. (TIF) [file pone.0177181.s002.tif]

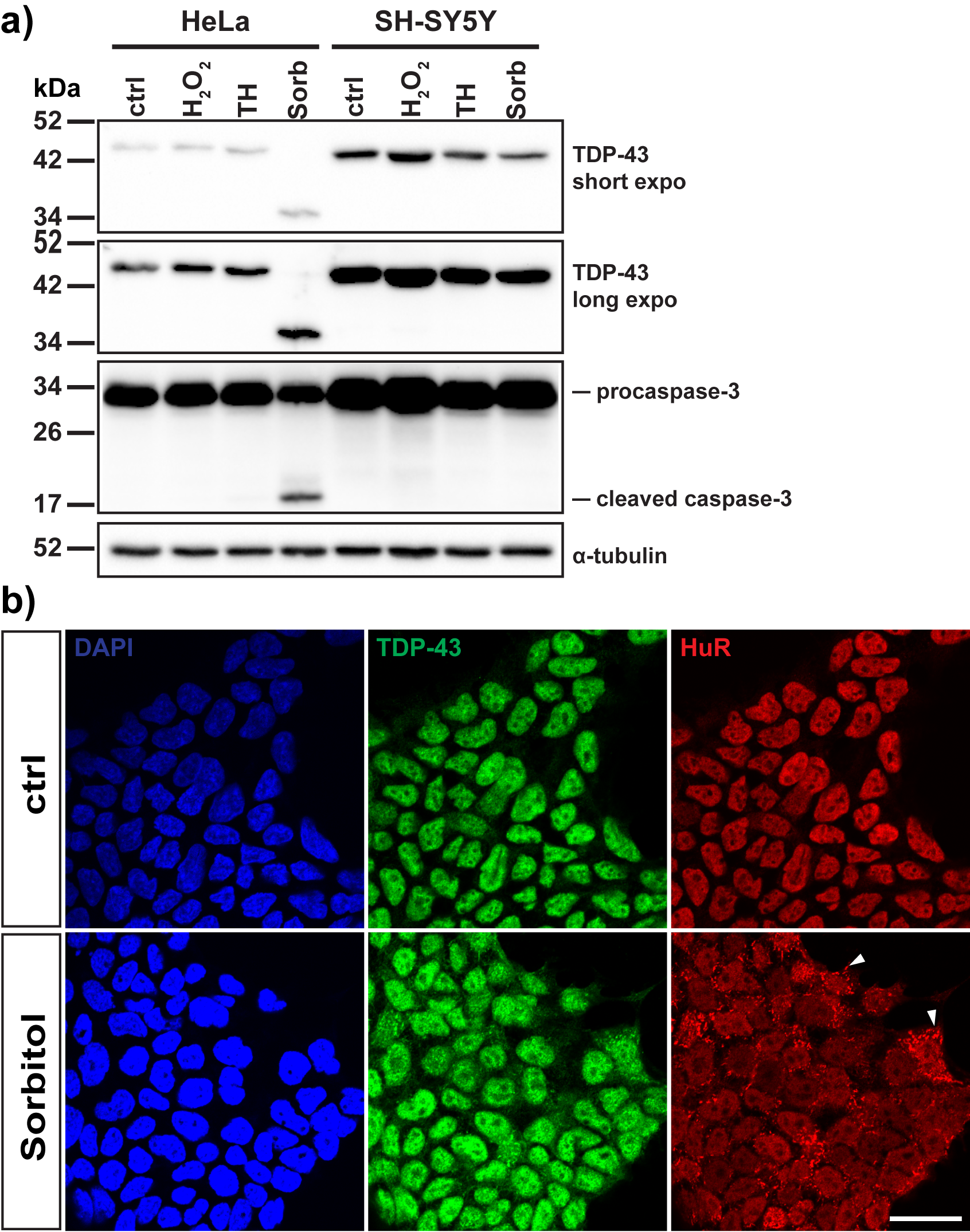

Supplement: S3 Fig — A) HeLa cells and undifferentiated SH-SY5Y cells were treated with 0.5 mM (SH-SY5Y) or 1 mM (HeLa) hydrogen peroxide (H2O2), 1 μM thapsigargin (TH) or 0.4 M D-sorbitol. The generation of CTF35 as well as the activation of caspase-3 was observed in HeLa cells after 1 hour of D-sorbitol treatment, but not in SH-SY5Y cells. B) Prominent formation of HuR-positive stress granules in SH-SY5Y cells in response to sorbitol (indicated with arrowhead). Blue = DAPI, green = TDP-43, red = HuR. Scale bar = 30 μm. (TIF) [file pone.0177181.s003.tif]

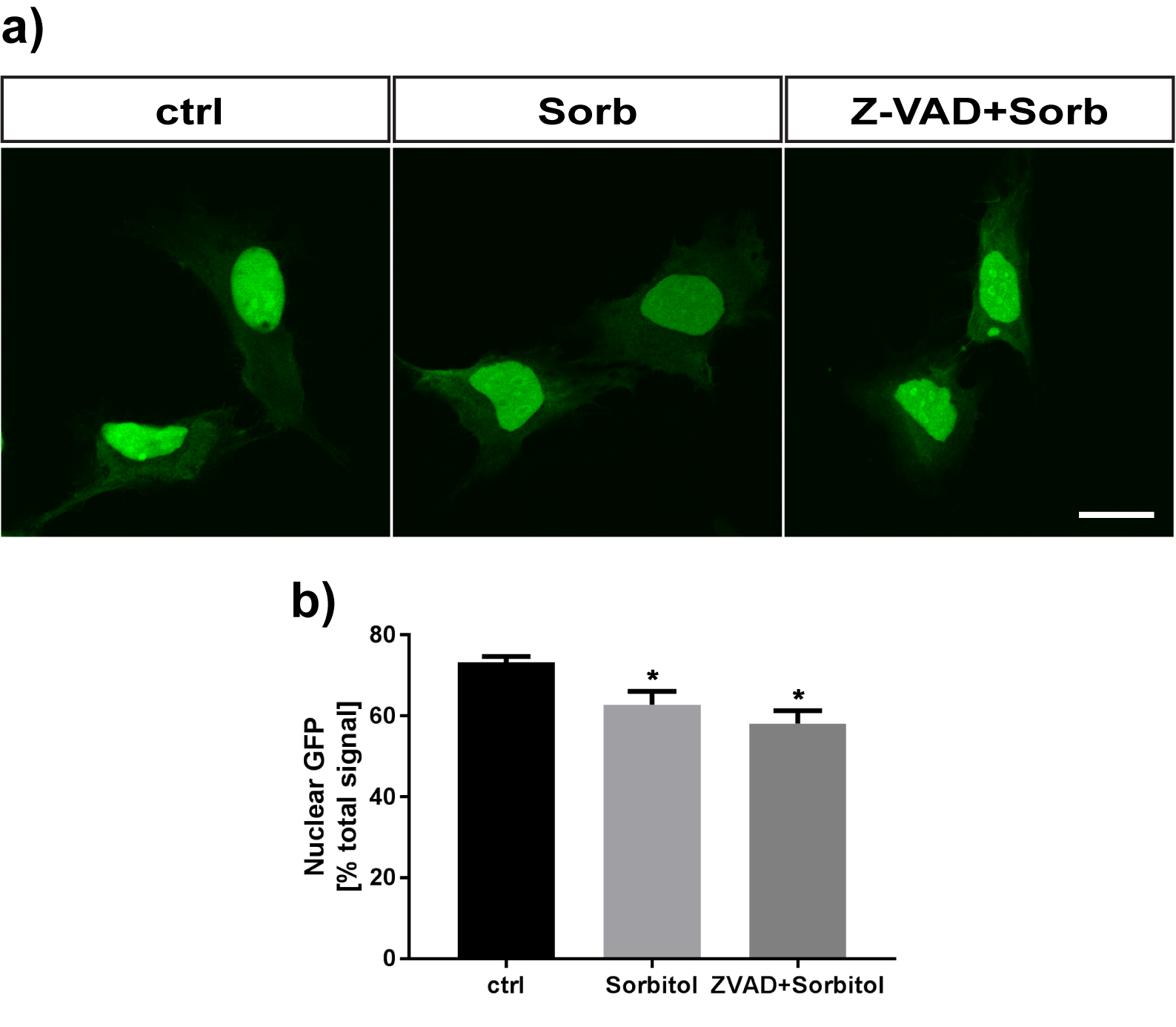

Supplement: S4 Fig — (A) Immunofluorescence images of HeLa cells transfected with EGFP fused to a nuclear localization sequence. After 24 h, cells were treated with DMSO or Z-VAD-FMK for 30 min, followed by regular medium or 0.4 M sorbitol for 60 min. Scale bar = 20 μm. (B) Quantification of nuclear NLS-EGFP signal as a proportion of total cellular EGFP signal (N = 3–4). Results represent mean percentage of nuclear GFP signal ± S.E.M.; * p < 0.05; one-way ANOVA (F2,8 = 7.754, P = 0.0134) followed by Dunnett’s multiple comparisons test. (TIF) [file pone.0177181.s004.tif]

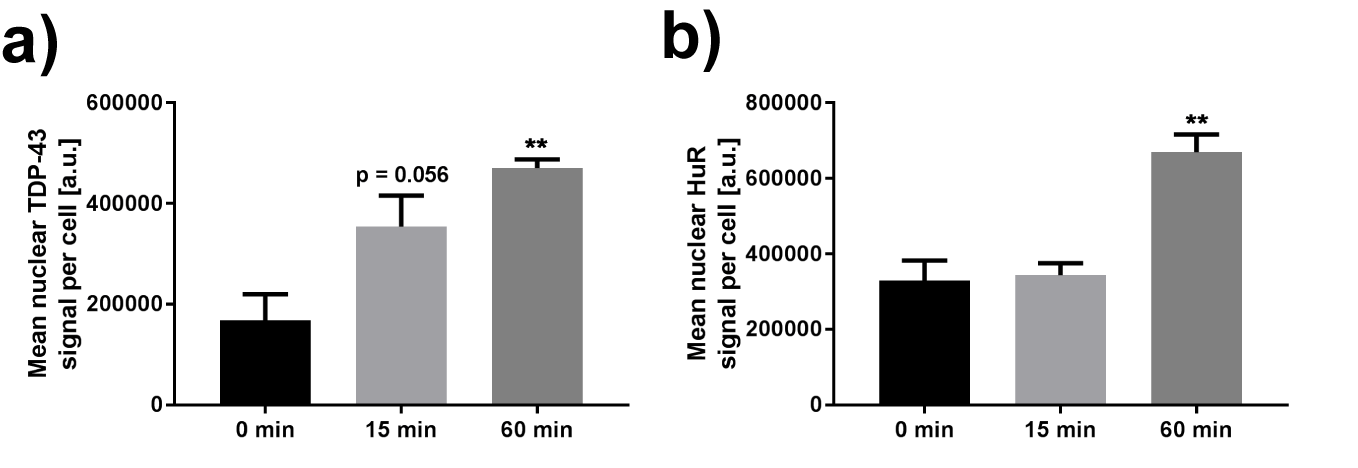

Supplement: S5 Fig — HeLa cells were exposed to 0.4 M D-sorbitol for 1 h, then left to recover for 0 min, 15 min or 60 min. (A) Nuclear TDP-43 and (B) HuR signals were quantified. Results represent mean nuclear immunofluorescence per cell ± SEM; ** p < 0.01 (N = 3); one-way ANOVA (TDP-43: F2,6 = 10.34, P = 0.0114; HuR: F2,6 = 18.72, P = 0.0026) followed by Dunnett’s multiple comparisons test. (TIF) [file pone.0177181.s005.tif]

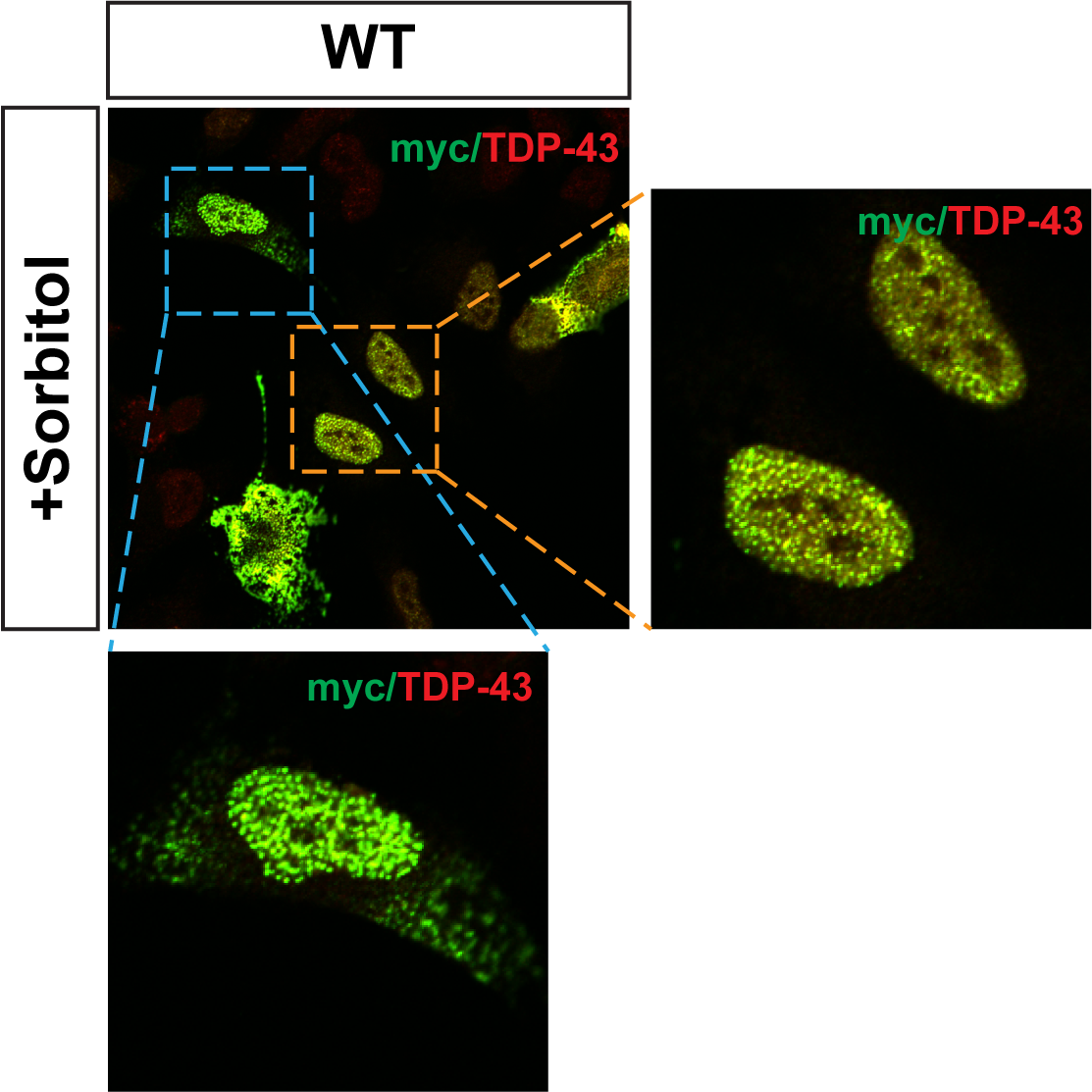

Supplement: S6 Fig — TDP-43 was expressed in HeLa cells for 24 h before sorbitol was added for 1 h. Overexpressed myc-TDP-43 forms a granular staining pattern in the nucleus indicative of protein aggregation both in cells with (blue rectangle) and without (orange rectangle) cytoplasmic TDP-43 relocalization (WT-transfected example). (TIF) [file pone.0177181.s006.tif]

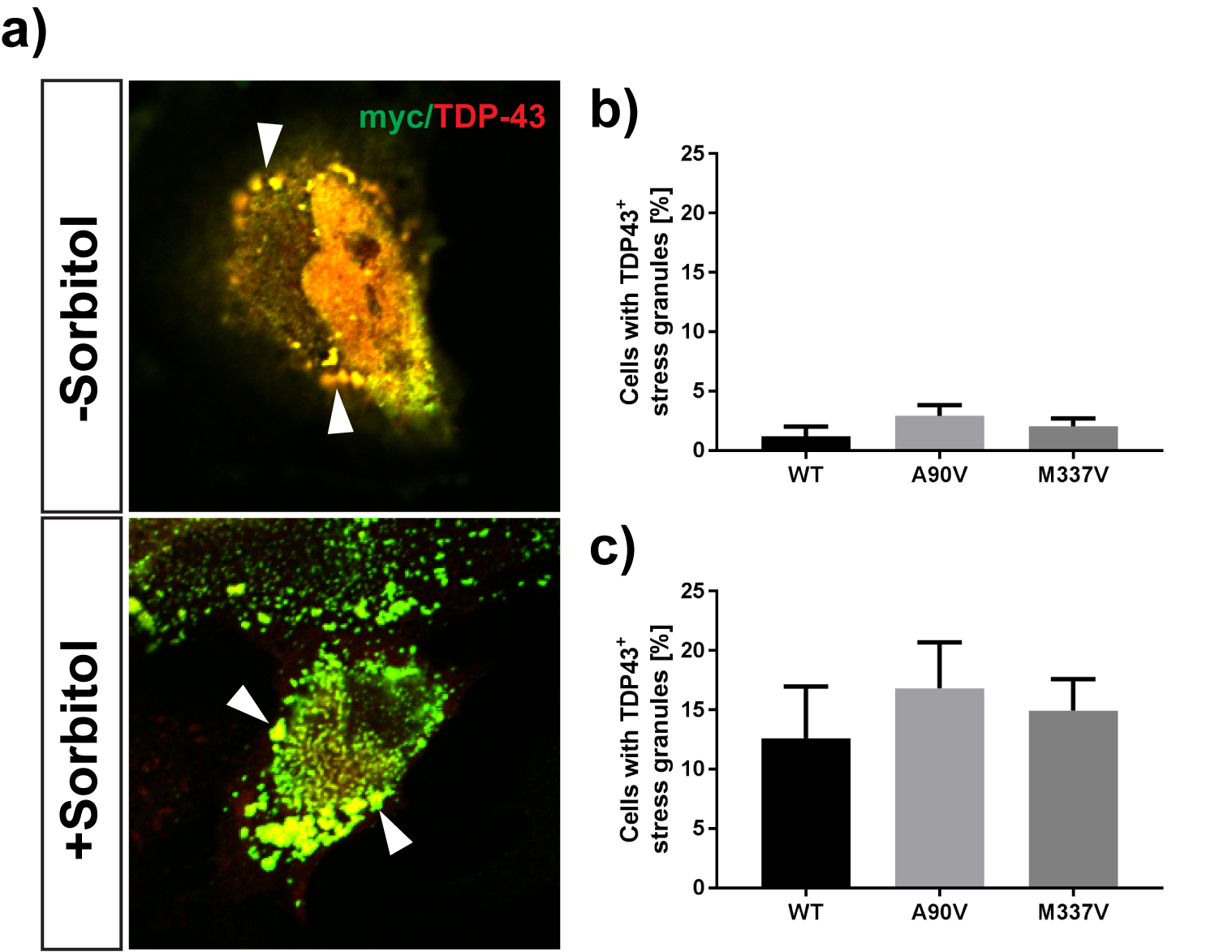

Supplement: S7 Fig — HeLa cells were transfected with WT, A90V or M337V TDP-43. After 24 h, cells were either left untreated or were treated with 0.4 M sorbitol for 1 h. (A) Immunofluorescence staining of transfected HeLa cells with or without additional sorbitol treatment. Green = myc, red = TDP-43. Arrowheads indicate stress granules. (B), (C) Quantification of proportion of transfected cells with TDP-43-positive stress granules in control conditions (B) or after sorbitol stress (C). Results represent mean percentage of transfected cells with TDP-43-positive cytoplasmic granules ± SEM. one-way ANOVA followed by Tukey’s multiple comparisons test (-Sorbitol: F2,12 = 1.098, P = 0.365; +Sorbitol: F2,12 = 0.3245, P = 0.729). (TIF) [file pone.0177181.s007.tif]
